# Eroded enamel rehardening using two intraoral appliances designs in different times of salivary exposure

**DOI:** 10.4317/jced.56222

**Published:** 2019-12-01

**Authors:** Fernanda-Lyrio Mendonça, Maisa-Camillo Jordão, Poliana-Pacífico Val, Catarina-Ribeiro-Barros de Alencar, Marcela-de Azevedo-Garcia Bassoto, Heitor-Marques Honório, Ana-Carolina Magalhães, Marília-Afonso-Rabelo Buzalaf, Thiago-Cruvinel da Silva, Daniela Rios

**Affiliations:** 1 Department of Pediatric Dentistry, Orthodontics and Public Health Bauru School of Dentistry, University of São Paulo - Bauru/SP - Brazil; 2 Department of Biological Sciences, Bauru School of Dentistry, University of São Paulo - Bauru/SP - Brazil

## Abstract

**Background:**

The aim of this study was evaluated the eroded enamel rehardening potential using upper palatal and lower buccal removable appliances in different times of salivary exposure (30 min, 1h, 2h, 12h) after a single erosive challenge event.

**Material and Methods:**

After initial surface hardness evaluation, bovine enamel blocks were eroded in vitro (0.01 M hydrochloric acid, pH 2.3, 30 seconds), selected (n = 160) and randomly assigned to the two appliance designs and twenty volunteers. Four enamel blocks were inserted in each removable appliance. On the in situ phase, the volunteers were instructed to use the upper palatal and lower buccal appliances simultaneously for 12 nonconsecutive hours. After each predetermined period of time of salivary exposure, the enamel blocks were removed from the appliances for immediate evaluation of surface hardness, enabling percentage of surface hardness recovery calculation (%SHR). The data were analyzed using two-way ANOVA and Tukey’s test (α=5%).

**Results:**

The results showed no difference in the degree of enamel rehardening by the upper palatal or lower buccal appliances (*p* >0.0001). Regarding the time of use of the appliances, it was demonstrated that 30 minutes (upper = 21.12%, lower = 19.84%) and 1 hour (upper = 35.69%, lower = 30.50%) promoted lower hardness recovery than two hours (upper = 44.65%, lower 40.80%) of salivary exposure (*p*<0.0001). The use of 12 hours (upper = 49.33%, lower = 49.00%), including the sleeping time of the volunteers did not increase the %SHR.

**Conclusions:**

The location of the appliance does not influence the re-hardening ability of saliva and the use of intraoral appliances for 2 hours seems to be appropriate for partial rehardening of the softened enamel surface.

** Key words:**Tooth erosion, in situ, saliva, tooth remineralization.

## Introduction

Saliva is an important biological factor for dental hard tissue health maintenance against dental erosion (1). It is a fluid secreted into the oral cavity by three pairs of major salivary glands: parotid, submandibular and sublingual (2). Due to its flow rate and inorganic/organic constituents, saliva can dilute, clear, neutralize and buffer the acids; enhance enamel mineral deposition by providing calcium, phosphate and fluoride, contributing to the repair of initial erosive lesion; and to reduce erosive demineralization by the formation of the acquired pellicle (1). Depending on the gland by which saliva is secreted, it may provide different levels of protection against tooth demineralization (3). In addition, sites poorly bathed by saliva are more susceptible to erosion (4), this means that labial surface of upper incisors is more likely to show erosion than lingual surface of lower teeth (5). Consequently, when considering *in situ* protocols for the study of dental erosion, the location of the appliance can influence the degree of tooth alteration.

Important scientific data regarding preventive measures for erosion comes from *in situ* models (6,7). The main advantage of the *in situ* models is the closely resembling of the natural environment due to the presence of salivary flow and acquired pellicle formation (8). They also enable the entire process of erosion to be monitored with accurate analytical methods of tissue loss measurement in the laboratory. However, little standardization of experimental *in situ* erosion models is available. Ideally, to mimic erosion susceptible sites, the specimens should be attached to facial, palatal and occlusal surfaces in maxilla (8). Nevertheless, the location of the specimens in such areas interferes with occlusion, not allowing this protocol. Different designs of intraoral appliances, which carry tooth specimens in the oral cavity, have been used in dental erosion *in situ* models (9,10). These appliances can be removable or fixed; on the lower or upper arches, with intermittent or continuous use (8).

Our research group have been studied different types of *in situ* appliances with a variety of time use, in order to find a protocol that better simulates a patient at risk of developing dental erosion (6,7,11-13). Santos *et al.* (6) investigated the effect of the period of use and location of intraoral appliances on enamel surface loss and have concluded that the intermittent use of appliances resulted in similar enamel loss compared to the continuous use. When considering all the erosive cycling process (erosive demineralization and rehardening), the enamel blocks located on the maxillary appliance presented significantly higher erosive enamel loss when compared to the enamel blocks of the mandibular appliance (6,7). On the other hand, when considering only one event of erosive demineralization there was no difference on the enamel hardness using the maxillary and mandibular appliances by volunteers (12) Another result showed no difference for the re-hardening effect after a single erosive demineralization (11), however is important to emphasize that in this last study the mandibular appliance was made with soft silicon plate, covering the entire lower arch, presenting a completely different design of the appliance used in the present study. Therefore, it is necessary to clarify using similar appliance design, if the higher enamel loss found when volunteers used palatal appliance under erosive cycling is due to less saliva ability to reharden in each cycle. This mechanism can be evaluated using the appliances a single time, immediately after erosive demineralization. However, the period of saliva effect, by the time of appliance use can influence on the re-hardening ability.

Taking this aspect into a consideration, the aim of this study was to compare the erosive enamel rehardening potential using upper palatal and lower buccal removable-appliances in different times of salivary exposure (30 min, 1h, 2h, 12h), in order to elucidate the mechanism in which there is a difference between the palatal and mandibular appliances in erosion cycling studies. The null hypotheses tested were that (1) there is no difference on eroded enamel rehardening potential when volunteers use upper palatal and lower buccal removable-appliances, and (2) there is no difference on eroded enamel rehardening potential when enamel is subjected to different times of salivary exposure, including overnight.

## Material and Methods

-Experimental Design

A single-blind (for analyst regarding type of removable-appliances), randomized, *in situ* experiment was designed. The experiment comprised one *in situ* period of 12 nonconsecutive hours. Twenty volunteers wore upper palatal and lower buccal appliances with 4 enamel blocks each, with a preformed initial erosion lesion. The factors under investigation were the type of removable appliance at 2 levels (upper and lower arches) and period of enamel *in situ* salivary exposition at 4 levels (30 minutes; 1 hour; 2 hours and 12 hours). The response variable was superficial hardness recovery. Superficial hardness was measured in the same enamel blocks at baseline, after erosion and after each period of salivary exposure.

-Ethical Approval

Ethical approval for the study was granted by the Local Research Ethics Committee (protocol no 2013/15765-2). This study was conducted in full accordance with the Declaration of Helsinki. Written informed consent was obtained from each volunteer at the beginning of the study, prior to confirmation of their eligibility for the study.

-Enamel samples preparation

Enamel blocks (4X4X3 mm, n=300) were prepared from the labial surfaces of bovine incisors crowns. The blocks were cut using ISOMET low speed saw cutting machine (Buehler Ltd., Lake Bluff, IL, USA) with two diamond disks (Extec Corp., Enfield, CT, USA), which were separated by a 4-mm thickness spacer. The blocks’ surfaces were ground flat with water-cooled silicon carbide discs (600 and 1200 grade papers; Buehler, Lake Bluff, IL, USA) and polished with felt paper wet by 1 µm diamond spray (Buehler, Ltd., Lake Bluff, IL, USA). The blocks were cleaned using an ultrasonic device for 10 min and firstly selected according to absence of white spots and cracks using a microscope (x40). The samples were sterilized using ethylene oxide.

The baseline surface hardness (SHi) was determined using the average values of five indentations performed at distances of 100 µm from each other (Knoop diamond, 25 g, 10 s, Hardness tester from Buehler, US). Two hundred eighty-nine blocks were selected according to the surface hardness values (SHi mean value = 358.93 ± 27.95 KHN) to be demineralized *in vitro* (initial erosion lesion). That is more than the number of blocks required, allowing the discharge of nonstandard demineralized blocks.

-Initial erosion lesion

Bovine enamel blocks were subjected to short-term acid exposure by immersion in hydrochloric acid (0.01M; pH 2.3) for 30 seconds under agitation (Flatbed oscillator, 60 rpm), resulting in surface softening without tissue loss. The surface hardness after demineralization was measured (SHd) at distances of 100 µm from the baseline surface hardness, to obtain the degree of softening. Enamel samples presenting the percentage of surface hardness change (%SHC = [(SHi – SHd) / (SHi)] x 100) between 30% and 40% were selected (n= 160) and randomly allocated to the volunteers and type of removable appliance by a second researcher using Microsoft Excel® 

-Inclusion and Exclusion Criteria

Twenty healthy adult volunteers (seventeen female and three males, aged 19–30 years) residing in the same fluoridated area (0.70 mg F/l) participated in the study, after satisfying the following inclusion criteria: physiological stimulated salivary flow rate (>1ml/min) and non-stimulated physiological salivary flow rate of >0.25 ml/min, adequate oral health with no caries, erosion lesions or significant gingivitis/periodontitis. The exclusion criteria were systemic illness, pregnancy or breastfeeding, under orthodontic intervention and use of professional fluoride compounds in the last two months.

Sample size calculation was based on a previous in situ pilot study. A sample size of 7 volunteers was estimated based on a α-error of 5%, β-error of 20%, 13.83% of superficial hardness recovery as estimated standard deviation and 30% as minimum detectable difference in means. Considering possible losses inherent to *in situ* studies, 20 volunteers were selected.

The removable upper palatal and lower buccal appliances were made for each volunteer with acrylic resin on the plaster model. The upper palatal appliance had two vertical rows, one on the right and the other on the left side, with two cavities (6x6x3 mm) in each side, for enamel blocks fixation (four blocks per appliance). In the case of the lowers buccal appliances there was only one row with two cavities (6x6x3 mm) for enamel blocks fixation (two blocks per appliance), however the volunteer used two appliances, one on the right and the other on the left side of the arch, fixed on first permanent molars by Adams clasp. The enamel blocks were fixed in the appliances with wax. An orthodontic wire was attached to the ends of the cavity passing over the enamel blocks without touching them, to prevent abrasion of the blocks by tongue or oral mucosa.

-*In situ* phase

Seven days prior to and during the experiment period, the volunteers brushed their teeth with standardized fluoride toothpaste (Total 12, 1,100 ppm F, Colgate, S.B. Campo, SP, Brazil). The volunteers were also warned to not use any other fluoride product. Toothbrushing with fluoride toothpaste was performed by the volunteers one hour prior to the insertion of intraoral appliances and initial of the experiment. The volunteers were instructed to use the maxillary and mandibular appliances containing eroded bovine enamel samples, for 12 nonconsecutive hours. Each volunteer wore one upper palatal and two lower buccal removable devices simultaneously. After a predetermined time of salivary exposure, corresponding to the group under study (30 minutes, 1 hour, 2 hours and 12 hours) volunteers removed the intraoral appliance (without washing) and the enamel blocks were subjected to surface hardness evaluation. A mean interval period of 4 hours was determined for hardness measurement and reposition of the enamel samples in their original cavities of the appliance, for the next period of *in situ* salivary exposition. Then, the volunteers wore the appliances on the maxilla and mandible until the next time under study was completed. This procedure was repeated until 12 hours of use was achieved; however, for the last evaluated period, the appliances were worn for 10 hours at night, during the volunteers’ sleep. For this reason, the volunteer and researcher blindness regarding the period of enamel in situ salivary exposition was not achievable. For the surface hardness measurements, special care was taken to avoid cross-contamination, including individualized use of gloves for handling each intraoral appliances and decontamination of Knoop diamond of Hardness tester after each measurement.

-Percentage of surface hardness recovery (SHR)

The final surface hardness (SHf) was measured at distances of 100 µm from the surface hardness after demineralization (SHd), as described above, for each studied time. The mean values of the five measurements were used to calculate the percentage of surface hardness recovery (%SHR = [(SHf-SHd) / SHi)] x100) for each block, then the mean value of the %SHR of 4 blocks for each volunteer (n = 20) in each arch was calculated.

-Statistical analysis

Statistical analysis was performed with SigmaPlot version 12.3 (2011 Systat Software, Germany). The assumptions of normal distribution of errors were checked using Shapiro-Wilk test. Since the assumptions were satisfied, repeated measures two-way ANOVA followed by Tukey’s test were applied. The significance level was set at 5%.

## Results

 Table 1 shows the mean values of the percentage of surface hardness recovery (%SHR) for the different times of salivary exposure and intraoral appliances (upper palatal and lower buccal).

Results showed no difference in the degree of enamel rehardening using upper palatal and lower buccal removable appliances. Given the periods of the intraoral devices use, 30 minutes promoted similar hardness recovery compared to 1 hour, and both resulted in lower rehardening compared to 2 h (*p*<0.0001). There was no statistically significant difference between 2 h and 12 h.

**Table 1:**
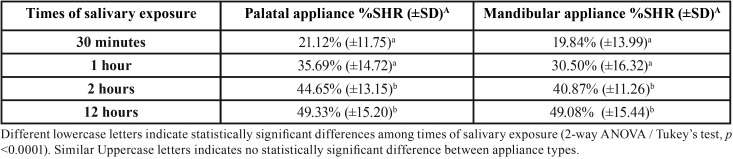
Mean and standard deviation values of the percentage of surface hardness recovery (%) for the studied times of salivary exposure using upper palatal and lower buccal removable appliances (n=20).

## Discussion

In enamel erosive lesions, mineral dissolution occurs on the surface area, resulting in a softened and roughened structure like an etching pattern (14). With the erosive challenge continuity or the incidence of mechanical forces, the increasingly softened layer is prone to bulk tissue loss (15). However, it has been speculated that the damage to the eroded softened layer could be reduced by remineralization (15). Moreover, recently it has been discussed that true remineralization does not occur on erosive lesions (14). According to Shellis *et al.* (16) the main obstacle to effective remineralization of *in vivo* erosive lesions is likely to be the low degree of supersaturation with respect to enamel minerals and the presence of inhibitors of calcium phosphate precipitation, such as salivary proteins. Scanning Electron Microscopy observation in one *in vitro* study showed an amorphous mineral deposition on top of enamel prisms, after 2 hours of remineralization (15). This is not an ideal form of mineralization, since the regrowth of the partly dissolved crystal is desired (16). In the present study, initial erosion lesions were developed *in vitro* to guarantee hardness measurements on the same surface, before acid exposure and after the *in situ* phase. The criterion used to ensure the stage of tissue softening without wear was the visualization of baseline indentations after short-term acidic exposure (17). Although clinically the rehardening of softened surface has little impact on erosion prevention, erosive cycling studies are driven by successive erosive challenges and it is essential to define the best interval between these challenges.

The current study helped to identify the time point between *in situ* erosive challenges with maximum rehardening potential of overnight oral conditions. The results showed that 30 minutes and 1 hour promoted lower hardness recovery than 2 hours of salivary exposure, which is similar to previous study (11). The use of 10 hours during the night did not increase the percentage of hardness recovery, confirming the results of Alencar *et al.* (13), who tested salivary exposure times for enamel rehardening *in situ* using only palatal removable appliances. Since salivary flow influences salivary parameters we hypothesize that this result may have been influenced by circadian salivary cycle, since at night the flow rate is low (18) negatively affecting the rehardening ability of saliva. Therefore, considering the remineralization process, overnight exposure to oral conditions does not incorporate beneficial effects of enamel rehardening into the *in situ* model. This result is in line with Santos *et al.* (6), in which the intermittent use of removable appliance behaved in the same manner as continuous, with the advantage that volunteers accepted better the use of easier protocols, allowing more realistic and reliable results.

In a previous *in vitro* study, the stabilization of surface-softened enamel was achieved by 6-24 hours of artificial saliva exposure, while a partial rehardening was obtained after 1-4 hours of artificial saliva exposure (15). However, although this result is related to a study *in vitro*, it knows that in clinical situations, intra-oral physical wear and the acid challenges limit the time for rehardening. In line with other studies, (15,19) our results showed that salivary exposure does not restore original surface hardness of sound enamel, recovering only 40% and 44% of the original hardness after 2 hours of salivary exposure. Fushida and Cury (20) showed up to 37.8% surface hardness recovery after 24 hours of *in situ* salivary exposure and the rehardening rate was significantly increased when specimens were treated with fluoride gel after the erosive attack. On the other hand, Austin *et al.* (21) found total hardness recovery *in vitro* by pooled human saliva after 6 hours.

In the present study, even with the use of fluoride dentifrice one hour before the positioning of the appliance in the oral cavity, the results obtained may be mainly due to the rehardening effect of saliva. Even though fluoride influences enamel remineralization, some studies have shown that the increase of fluoride in saliva after using a fluoride dentifrice is usually very limited and of short duration (22,23). In addition, residual salivary fluoride from dentifrice does not present any protective effect against erosion (24).

Different sites in the oral cavity can render teeth more susceptible to erosion because of their exposure to mechanical forces resulting from soft tissues (25) and tongue (26). In the present study, retaining wires were used onto the enamel blocks to avoid the abrasive effect of soft tissues and tongue. Prevention of enamel wear was fundamental for the study design, because the hardness recovery is only valid when the same surface is evaluated. Previous studies have not used (11,13) or used different methods to avoid the influence of abrasion on the samples such as the placement of retaining wires (27) or the fixation of samples 0.5 mm below the surface contacting (25,28).

Submandibular gland is the major contributor to the unstimulated salivary flow, which produces saliva with high concentrations of mucin. On the other hand, parotid flow increases dramatically during stimulation, and its main role is related to the buffer capacity (2). Furthermore, with the salivary stimulation, there is an increase in salivary flow and the amount of calcium and phosphate, which could benefit rehardening (1). Removable appliances behave as a mechanical stimulus to increase salivary flow in *in situ* studies. In the design of the mandibular appliances, enamel blocks were in the buccal region of the second premolar and first permanent molar. This region is close to the parotid glands. According to Lussi *et al.* (29), faster pH recovery was observed after ingestion of orange juice on the second mandibular premolar compared to the maxillary central incisor. The authors suggested that this may have been due to the proximity of the tooth with the parotid gland. Thus, it was expected that the enamel samples in the mandibular appliances, supposedly under greater influence of the parotid gland, could be benefited with increased salivary flow and subsequent remineralization. However, this study showed no difference in enamel remineralization degree using upper palatal and lower buccal appliances, therefore the re-hardening effect of saliva from different sites might not be the responsible for the less enamel loss resulted from the use of mandibular appliance in previous studies (6,7).

## Conclusions

In conclusion, considering only eroded enamel rehardening potential, the location of the appliance does not influence the re-hardening ability of saliva and the use of intraoral appliances for 2 hours seems to be appropriate for partial rehardening of the softened enamel surface. Therefore, the use of intraoral appliance overnight is not justified for rehardening protocols, suggesting that the use of simple protocols may be better accepted by the volunteers, allowing more realistic and reliable results. Considering these aspects, the first null hypothesis was accepted and the second was rejected.
